# Physical Health Monitoring of Patients Prescribed Depot Antipsychotic Medication and Clozapine in the North-West Edinburgh Community Mental Health Team

**DOI:** 10.1192/bjo.2025.10505

**Published:** 2025-06-20

**Authors:** Andrew Revadillo, Anna Lunan, Jack Mitchell, Kenneth Murphy, Douglas Murdie

**Affiliations:** 1 University of Edinburgh Medical School, Edinburgh, United Kingdom; 2 NHS Lothian, Edinburgh, United Kingdom

## Abstract

**Aims:** Antipsychotic medications are associated with metabolic syndrome and increased cardiovascular risk. Monitoring the physical health of patients receiving these medications is a key part of delivering safe and effective care.

Since 2020, audit cycles in the North-West Edinburgh Community Mental Health Team (NWCMHT) have found this monitoring to be consistently poor.

An experienced nurse was appointed lead of a new physical health clinic and it was incorporated into timetables of junior doctors to facilitate liaison with primary care. This weekly clinic was established in NWCMHT in 2023 focusing on those prescribed depot antipsychotic medication and clozapine.

We aim to assess the impact of this service development on patient care.

**Methods:** Scottish Intercollegiate Guidelines Network publication 131 was used as the gold standard, which cites 9 domains to monitor annually – past medical history (PMH), family history (FH), smoking history, BMI (or weight or waist circumference), blood pressure (BP), HbA1c, lipids, prolactin and ECG.

Data was collected for these domains for patients prescribed and administered depot antipsychotic medication or clozapine in the NWCMHT for the calendar year of 2024. Data was collected from the local computerised clinical notes system (TRAK) and anonymised in line with NHS Information Governance Policy.

**Results:** 163 patients were prescribed depot antipsychotic medication or clozapine by the NWCMHT in 2024. 58% (n=95) of these patients were offered an appointment at the physical health clinic, with 37% attending (n=60).

Across all domains, monitoring of those who attended clinic was better than those who did not – PMH (97% vs 48%), FH (95% vs 36%), Smoking (95% vs 44%), BMI (87% vs 28%), BP (97% vs 69%), HBA1c (82% vs 55%), lipids (74% vs 49%), prolactin (51% vs 35%) and ECG (85% vs 36%).

Of all 163 patients, the average completed monitoring across all nine domains was 60% in 2024. The average across all domains before the clinic was established was 30%.

**Conclusion:** There has been a significant improvement in monitoring in this patient group since the clinic was established in 2023. Patients who attend this clinic are monitored more effectively.

However, there are opportunities for further improvement. This would include identifying barriers that arise in achieving 100% across all domains in attendees and assessing factors that impede attendance at the clinic.

These results support plans to expand the clinic to ensure that the physical health of this patient group is appropriately monitored to achieve safe and effective care.

